# Erythropoietic protoporphyria life impact and genetic health trajectory (LIGHT) study in Europe: a cross-sectional online questionnaire

**DOI:** 10.1186/s13023-026-04572-3

**Published:** 2026-08-27

**Authors:** Hetanshi Naik, Kristen Wheeden, Susan D. Mathias, Chelsea Norregaard, Adaeze Q. Amaefule, Melanie Chin, Manisha Balwani

**Affiliations:** 1 Department of Genetics, Stanford University School of Medicine, 453 Quarry Road, Palo Alto, Stanford, CA 94304 USA; 2 United Porphyrias Association, Bethesda, MD USA; 3 Health Outcomes Solutions, Palm Beach Gardens, FL USA; 4 Disc Medicine, Watertown, MA USA; 5 Department of Genetics and Genomic Sciences, Icahn School of Medicine at Mount Sinai, New York, NY USA

**Keywords:** Erythropoietic protoporphyria, Quality of life, Patient-reported outcomes, Healthcare utilization, Phototoxicity

## Abstract

**Background:**

Erythropoietic protoporphyria and X-linked protoporphyria (collectively, EPP) are rare inherited metabolic disorders of the heme biosynthetic pathway characterized by severe photosensitivity and painful phototoxic reactions upon sunlight exposure. This study sought to describe health-related quality of life, symptoms, and healthcare resource utilization in European patients with EPP. Participants aged ≥ 12 years with a diagnosis of EPP residing in France, Germany, Italy, Spain, or the UK completed a one-time online questionnaire. Patient consent and ethics approval were obtained.

**Results:**

Ninety-one adults and 10 adolescents participated; 76% of adults and 38% of adolescents could spend ≤ 30 minutes in direct or indirect sunlight before prodromal symptoms started. Thirty-six percent (Italy) to 67% (Spain) of participants experienced prodromal symptoms ≥ 5 times in the past three months. Sixty-nine percent of participants experienced pain after < 30 minutes of direct sunlight, and over half experienced pain from indirect sunlight. The most recent phototoxic reaction limited daily activities “very much” in 58% of participants (range: 40% Spain to 86% UK). Participants reported a high emotional burden, including sadness/depression (66%), anxiety (60%), frustration (86%), loneliness (74%), and isolation (84%) due to their EPP. EPP negatively impacted work productivity for 9% (Italy) to 42% (France) of participants currently working.

**Conclusions:**

Europeans with EPP frequently experience prodromal symptoms and phototoxic reactions that impose a negative impact on health-related quality of life and ability to do daily activities, highlighting the need for early diagnosis and effective treatments.

**Supplementary Information:**

The online version contains supplementary material available at https://doi.org/10.1186/s13023-026-04572-3.

## Background

Erythropoietic protoporphyria and X-linked protoporphyria (collectively referred to as EPP^1^) are rare genetic disorders of the heme biosynthetic pathway [1, 2]. EPP is a debilitating condition characterized by skin phototoxicity to sunlight (both direct and indirect) and to some types of artificial light (e.g., fluorescent light). Symptoms after exposure to sunlight or artificial light include burning, itching, and pain on exposed areas of the skin [2–5], which can last for hours or even days [5]. Affected individuals may also experience edema and/or redness or inflammation of the skin [2]. Areas not typically covered in clothing, such as the dorsum of the hands, arms, and face, are most commonly affected.

Studies from European countries estimate EPP prevalence to be 1 in 57,000 to 1 in 200,000 [6–8]. The onset of photosensitivity generally occurs in early childhood [2]. The diagnosis of EPP is often delayed, with a US study reporting the mean time between initial symptoms and diagnosis of 13 years [9]. The health-related quality-of-life (HRQoL) impact for individuals with EPP is significant [10–13].

Studies of individuals with EPP living in Sweden, the UK, and the Netherlands have reported that EPP imposes significant psychosocial strain and decrements to HRQoL from pain, poorer physical functioning, and worse mental and emotional health [13–15]. However, information regarding the burden of EPP on children and healthcare resource use (HRU) in Europe is lacking. There are limited treatment options available for EPP; therefore, management often requires lifestyle modifications such as sunlight avoidance. There remains an underlying need for new and effective treatments.

Therefore, this study sought to describe symptoms, impacts to HRQoL (including physical, mental, emotional, and social functioning), and HRU associated with EPP, as well as satisfaction with and preference for treatments among individuals in France, Germany, Italy, Spain, and the UK.

## Methods

### Participants and study type

Individuals aged 12 years or older with a self-reported diagnosis of EPP who resided in France, Germany, Italy, Spain, or the UK, were recruited through local EPP patient advocacy groups (Selbsthilfe EPP e.V. [Germany], Association Française des Malades Atteints de Porphyries [France], Vivi Porfiria ODV, the British Porphyria Association [UK], and Asociación Española de Porfiria [Spain]). The countries of France, Germany, Italy, Spain, and the United Kingdom were selected for inclusion because they constitute the EU5 countries, and are of primary interest for healthcare decision-making, market access, and reimbursement assessments. Data collection took place from August to October 2025.

Eligible participants were required to speak, read, and write in English, French, German, Italian, Spanish, or Welsh and be willing to complete one online questionnaire that could take approximately 60 minutes. Those with a medical or psychiatric condition or treatment for a condition that resulted in a cognitive or other (e.g., visual, hearing) impairment that would interfere with participating in the study were excluded. Clinical study nurses in each participating country (except France) screened interested individuals to ensure they met the eligibility. Data protection laws in France made the use of nurse screening not feasible, so self-reporting of an EPP diagnosis was used for individuals in France.

Participants (or their caregiver/parent when appropriate) provided consent to the use of personal data for this study, except in France, where non-opposition was implied by the completion of the study questionnaire. Independent ethics approvals were obtained in the countries that required them: Spain, Italy, and the UK.

### Questionnaires and data collection

The questionnaire contained several existing, validated, and original patient-reported outcome (PRO) HRQoL measures. These included questions from the EPP Impact Questionnaire (EPIQ) [16], questions assessing prodromal symptoms and pain from EPP (Sunlight Exposure Diary) [17], select domains from the KIDSCREEN-27 Psychological Well-Being and Peers and Social Support (adolescents only) [18, 19], and Patient-Reported Outcomes Measurement System (PROMIS) scales (Satisfaction with Social Roles and Activities − 8a, Peer Relationships − 8a [adolescents only], and Social Isolation − 4a) [20]. The questionnaire defined prodrome as “early warning symptoms,” and participants self-selected symptoms they associated with their prodrome (if they reported experiencing one). The questionnaire defined a phototoxic reaction as the full reaction following prodromal symptoms that typically involved pain, swelling, burning and other symptoms. The questionnaire also included original questions assessing physical functioning, anxiety, depression, fatigue, bullying/being treated unfairly, work/school absenteeism or productivity loss, and need for accommodations in doing daily activities. Additional original questions assessing EPP diagnosis and HRU, such as visits to the emergency room (ER) or hospital, visits to physicians, and use of both prescription and over-the-counter medication use were also included. Other original questions were included to assess experience with and preference for various modes of treatment administration (e.g., oral versus implant). Finally, participants also completed questions assessing demographic and clinical characteristics for describing the study population.

Two versions of the questionnaire were developed: one for adults and one for adolescents. These were translated into each local language for use in each of the five countries.

### Statistical analyses

Data were analyzed descriptively. There were no pre-specified hypotheses, and therefore, no formal hypothesis testing was conducted. Scale scores were calculated for the existing PRO measures using established scoring guidelines and original questions were analyzed individually. For the PROMIS measures, T-scores were calculated for each participant, as detailed by those measures’ instructions. The T-score is a standardized score, with a mean of 50 and a standard deviation (SD) of 10. On the PROMIS Social Isolation measure, a higher score represents more isolation, while on the PROMIS Satisfaction with Social Roles and Activities measure, a lower score reflects less satisfaction. A lower score on the PROMIS Peer Relationships-8a represents a worse relationship with peers. A difference of three T-score points or more is considered meaningful on PROMIS measures [21, 22].

For the KIDSCREEN-27, T-scores were calculated for the two domains: Peers and Social Support and Psychological Well-Being. For each domain, a higher *T*-value represents better HRQoL. KIDSCREEN-27 does not provide information on differences in T-scores that are considered meaningful, so for this study it was assumed that a difference of at least 0.5 standard deviations (or 5 T-score points) reflects a meaningful difference.

### Compensation and ethical considerations

Each participant received a £80 / €95 shopping voucher upon completion of the questionnaire. This study was submitted to ethical review boards for approval or waivers whenever required by local law. Regulatory authorities were notified, and approval was sought as required by local laws and regulations.

## Results

### Demographic and clinical characteristics

Ninety-one adult and 10 adolescent participants completed the questionnaire (5 adults and no adolescents had confirmed X-linked protoporphyria), including 33 from France, 21 from Germany, 15 from Italy, 10 from Spain, and 22 from the UK. France had the most adolescent participants (6), followed by the UK (3) and Spain (1); there were no adolescent participants from Italy or Germany. The majority of adults was female (69%), with a college degree or higher (58%), married (51%), and working full- or part-time for pay (82%). Fifty percent of adolescents were female, 60% had a high school diploma or equivalent, and 100% went to school full-time. The youngest participants, on average, were from France (mean 37.1 years); the oldest participants were from Italy (mean 50.3 years). Females outnumbered males in each country, but marital status, income, and work/school status varied by country (Table 1).

**Table 1:** Sociodemographic and clinical characteristics of full sample by European country

Characteristic	France (n = 33)	Germany (n = 21)	Italy (n = 15)	Spain (n = 10)	UK (n = 22)
Age, years, mean±SD (range)	*n =* 32	*n =* 20			
	37.1 ± 18.7 (14–87)	48.1 ± 12.5 (18–68)	50.3 ± 11.2 (28–68)	39.6 ± 14.5 (13–54)	43.5 ± 18.0 (12–79)
Gender, n (%)					
Male	10 (30.3)	8 (38.1)	5 (33.3)	2 (20.0)	6 (27.3)
Female	22 (66.7)	12 (57.1)	10 (66.7)	8 (80.0)	16 (72.7)
Non-binary	1 (3.0)	1 (4.8)	0 (0.0)	0 (0.0)	0 (0.0)
Education, n (%)					
Middle school/junior high	4 (12.1)	5 (23.8)	0 (0.0)	1 (10.0)	2 (9.1)
High school diploma or equivalent	7 (21.2)	7 (33.3)	8 (53.3)	1 (10.0)	7 (31.8)
Some college	1 (3.0)	3 (14.3)	0 (0.0)	1 (10.0)	1 (4.6)
College degree	12 (36.4)	5 (23.8)	7 (46.7)	7 (70.0)	6 (27.3)
Professional/advanced degree	9 (27.3)	1 (4.8)	0 (0.0)	0 (0.0)	6 (27.3)
Marital status, n (%)	*n =* 27	*n =* 21	*n =* 14	*n =* 9	*n =* 19
Married	11 (40.7)	11 (52.4)	10 (71.4)	3 (33.3)	11 (57.9)
Living together with a partner	6 (22.2)	4 (19.1)	1 (7.1)	1 (11.1)	1 (5.3)
Widowed	0 (0.0)	1 (4.8)	1 (7.1)	0 (0.0)	0 (0.0)
Divorced	2 (7.4)	0 (0.0)	1 (7.1)	1 (11.1)	1 (5.3)
Separated	2 (7.4)	1 (4.8)	1 (7.1)	0 (0.0)	1 (5.3)
Single/Never married	6 (22.2)	4 (19.1)	0 (0.0)	4 (44.4)	5 (26.3)
Annual household income, n (%)	*n =* 21	*n =* 16	*n =* 11	*n =* 7	*n =* 18
< £15,000	3 (14.3)	1 (6.3)	0 (0.0)	1 (14.3)	2 (11.1)
£15,000 to 29,999	9 (42.9)	2 (12.5)	5 (45.5)	2 (28.6)	2 (11.1)
£30,000 to 49,999	3 (14.3)	0 (0.0)	3 (27.3)	1 (14.3)	4 (22.2)
£50,000 to 74,999	4 (19.1)	7 (43.8)	3 (27.3)	2 (28.6)	4 (22.2)
≥ £75,000	2 (9.5)	6 (37.5)	0 (0.0)	1 (14.3)	6 (33.3)
Work/school status, n (%)	*n =* 32	*n =* 21	*n =* 14	*n =* 10	*n =* 22
Work full-time for pay	21 (65.6)	12 (57.1)	10 (71.4)	7 (70.0)	10 (45.5)
Work part-time for pay	2 (6.3)	5 (23.8)	2 (14.3)	0 (0.0)	7 (31.8)
Do not work due to EPP	0 (0.0)	0 (0.0)	0 (0.0)	0 (0.0)	0 (0.0)
Do not work, unrelated to EPP	1 (3.1)	2 (9.5)	1 (7.1)	1 (10.0)	2 (9.1)
Go to school part time	0 (0.0)	1 (4.8)	0 (0.0)	0 (0.0)	0 (0.0)
Go to school full time	6 (18.8)	1 (4.8)	0 (0.0)	2 (20.0)	3 (13.6)
Other	6 (18.8)	5 (23.8)	1 (7.1)	0 (0.0)	1 (4.6)

The mean age at the time of diagnosis for the full sample was 17 years, and most (67%) diagnoses were confirmed based on genetic testing. Fifty-one percent of participants reported the time to EPP diagnosis was ≥ 5 years, but that varied by country: that percentage was fewer than half in France (36%), Spain (40%), and the UK (41%), but much higher in Italy (73%) and Germany (76%). Co-morbid conditions were common among participants and included vitamin D deficiency (71% of adults, 70% of adolescents), anemia (55% of adults, 40% of adolescents), and gallstones (30% of adults, 0% of adolescents). Eight adults (9%) and 1 adolescent (10%) reported liver disease at the time of the study (defined as elevated liver enzymes or liver fibrosis). Seventeen percent of participants (all adults) had their gallbladder removed, and 79% (81% of adults and 60% of adolescents) had undergone a liver ultrasound.

### Prodromal symptoms and phototoxic reactions

Seventy-six percent of adults and 38% of adolescents indicated that they could spend ≤ 30 minutes in direct or indirect sunlight before having prodromal symptoms. Within each country, the overall percent that experienced prodromal symptoms after ≤ 30 minutes ranged from 67% in Germany to 81% in the UK (Table 2). Half of participants (50% of adults and 50% of adolescents; Supplemental Table S1) experienced prodromal symptoms at least five times in the past three months (range: 36% in Italy to 67% in Spain). All adolescent participants (100%) and 44% of adults said prodromal symptoms affect their ability to do daily activities “quite a bit” or “very much.” That percent in the full sample was higher in France (63%), Spain (70%), and the UK (57%) than in Italy (43%) and Germany (21%). In all countries, a majority reported that they “usually” or “always” experience prodromal symptoms before a phototoxic reaction (range: 71% in Italy − 90% in Spain).

**Table 2:** Timing and characteristics of prodromal symptoms, by European country

Characteristic	France (n = 33)	Germany (n = 22)	Italy (n = 15)	Spain (n = 10)	UK (n = 22)
Time in sunlight before having prodromal symptoms, n (%)	*n =* 31	*n =* 21	*n =* 14	*n =* 9	*n =* 21
≤10 minutes	9 (29.0)	7 (33.3)	3 (21.4)	1 (11.1)	6 (28.6)
>10 to ≤30 minutes	12 (38.7)	7 (33.3)	7 (50.0)	6 (66.7)	11 (52.4)
>30 minutes	10 (32.3)	7 (33.3)	4 (28.6)	2 (22.2)	3 (14.3)
Times in past 3 months experienced prodromal symptoms, n (%)	*n =* 32	*n =* 21	*n =* 14	*n =* 9	*n =* 20
0 times	4 (12.5)	2 (9.5)	3 (21.4)	1 (11.1)	3 (15.0)
5 to 9 times	14 (43.8)	7 (33.3)	6 (42.9)	2 (22.2)	6 (30.0)
10+ times	4 (12.5)	1 (4.8)	2 (14.3)	2 (22.2)	2 (10.0)
1 to 4 times	10 (31.3)	11 (52.4)	3 (21.4)	4 (44.4)	9 (45.0)
Did prodromal symptoms affect ability to do daily activities, n (%)	*n =* 32	*n =* 19	*n =* 14	*n =* 10	*n =* 21
Not at all	1 (3.1)	1 (5.3)	0 (0.0)	0 (0.0)	1 (4.8)
A little bit	4 (12.5)	6 (31.6)	5 (35.7)	1 (10.0)	4 (19.0)
Somewhat	7 (21.9)	8 (42.1)	3 (21.4)	2 (20.0)	4 (19.0)
Quite a bit	13 (40.6)	4 (21.1)	4 (28.6)	6 (60.0)	3 (14.3)
Very much	7 (21.9)	0 (0.0)	2 (14.3)	1 (10.0)	9 (42.9)
How often experience prodromal symptoms before a phototoxic reaction, n (%)	*n =* 31	*n =* 21	*n =* 14	*n =* 10	*n =* 20
Never	0 (0.0)	1 (4.8)	0 (0.0)	0 (0.0)	0 (0.0)
Rarely	1 (3.2)	1 (4.8)	0 (0.0)	0 (0.0)	0 (0.0)
Sometimes	6 (19.4)	2 (9.5)	4 (28.6)	1 (10.0)	3 (15.0)
Usually	8 (25.8)	4 (19.5)	0 (0.0)	2 (20.0)	11 (55.5)
Always	16 (51.6)	13 (61.9)	10 (71.4)	7 (70.0)	6 (30.0)

The most common prodromal symptom reported for the full sample was sensitivity to hot/cold (84%; range = 71% in Italy to 91% in Germany), followed by sensitivity to touch (83%; range = 75% in UK to 90% in Spain), feelings of warmth (78%; range = 57% in Italy to 84% in France), burning (70%; range = 50% in Italy to 90% in Spain), itching (69%; range = 57% in Italy to 90% in Spain), and pain (68%; range = 50% in Italy to 80% in Spain). In the full sample, of those who experienced prodromal symptoms of tingling, burning, sensitivity to hot or cold, sensitivity to touch, pain, feelings of warmth, and swelling, ≥50% reported the severity as “moderate” or “severe.”

More than half (53%) of participants reported they “often” or “always” experience pain from indirect sunlight (range = 50% Spain and UK to 60% Germany; Table 3), and 58% experience pain from a phototoxic reaction after < 30 minutes in indirect sunlight (range = 52% France to 73% UK). When exposed to direct sunlight, 69% reported experiencing pain from a phototoxic reaction after < 30 minutes (range = 60% Italy to 82% UK).

**Table 3:** Sunlight exposure and phototoxic reactions, by European country

Characteristic	France (n = 33)	Germany (n = 21)	Italy (n = 15)	Spain (n = 10)	UK (n = 22)
Phototoxic reactions in past 12 months, n (%)	*n =* 32	*n =* 21	*n =* 15	*n =* 9	*n =* 22
0	13 (40.6)	7 (33.3)	6 (40.0)	3 (33.3)	7 (31.8)
1 to 3	17 (53.1)	10 (47.6)	8 (53.3)	4 (44.4)	13 (59.1)
4 to 9	1 (3.1)	4 (19.1)	1 (6.7)	2 (22.2)	2 (9.1)
≥10	1 (3.1)	0 (0.0)	0 (0.0)	0 (0.0)	0 (0.0)
Time since last phototoxic reaction in years, n (%)	*n =* 33	*n =* 21	*n =* 14	*n =* 10	*n =* 22
<1 year	12 (36.4)	9 (42.9)	6 (42.9)	6 (60.0)	14 (63.6)
1 to 3 years	13 (39.4)	5 (23.8)	3 (21.4)	3 (30.0)	5 (22.7)
4 to 9 years	3 (9.1)	3 (14.3)	1 (7.1)	0 (0.0)	0 (0.0)
≥10 years	5 (15.2)	4 (19.1)	4 (28.6)	1 (10.0)	3 (13.6)
How often pain from indirect sunlight, n (%)	*n =* 33	*n =* 20	*n =* 15	*n =* 10	*n =* 22
Never	2 (6.1)	1 (5.0)	0 (0.0)	0 (0.0)	1 (4.6)
Rarely	6 (18.2)	4 (20.0)	1 (6.7)	3 (30.0)	4 (18.2)
Sometimes	8 (24.2)	3 (15.0)	6 (40.0)	2 (20.0)	6 (27.3)
Often	10 (30.3)	7 (35.0)	3 (20.0)	3 (30.0)	6 (27.3)
Always	7 (21.2)	5 (25.0)	5 (33.3)	2 (20.0)	5 (22.7)
Minutes in indirect sun before pain from phototoxic reaction, n (%)	*n =* 33	*n =* 19	*n =* 15	*n =* 10	*n =* 22
<5 minutes	2 (6.1)	2 (10.5)	0 (0.0)	0 (0.0)	2 (9.1)
5 to <10 minutes	5 (15.2)	2 (10.5)	3 (20.0)	0 (0.0)	4 (18.2)
10 to <30 minutes	10 (30.3)	6 (31.6)	5 (33.3)	6 (60.0)	10 (45.5)
30 to <60 minutes	11 (33.3)	1 (5.3)	3 (20.0)	1 (10.0)	3 (13.6)
60 or more minutes	5 (15.5)	8 (42.1)	4 (26.7)	3 (30.0)	3 (13.6)
Minutes in direct sun before pain from phototoxic reaction, n (%)	*n =* 32	*n =* 21	*n =* 15	*n =* 10	*n =* 22
<5 minutes	6 (18.8)	3 (14.3)	3 (20.0)	1 (10.0)	5 (22.7)
5 to <10 minutes	6 (18.8)	5 (23.8)	3 (20.0)	4 (40.0)	4 (18.2)
10 to <30 minutes	10 (31.6)	5 (23.8)	3 (20.0)	2 (20.0)	9 (40.9)
30 to <60 minutes	6 (18.8)	5 (23.8)	2 (13.3)	2 (20.0)	4 (18.2)
60 or more minutes	4 (12.5)	3 (14.3)	4 (26.7)	1 (10.0)	0 (0.0)
How last phototoxic reaction affected ability to do daily activities, n (%)	*n =* 32	*n =* 21	*n =* 14	*n =* 10	*n =* 22
Not at all	0 (0.0)	0 (0.0)	0 (0.0)	0 (0.0)	0 (0.0)
A little bit	1 (3.1)	0 (0.0)	0 (0.0)	0 (0.0)	1 (4.6)
Somewhat	1 (3.1)	2 (9.5)	3 (21.4)	0 (0.0)	1 (4.6)
Quite a bit	12 (37.5)	9 (42.9)	5 (35.7)	6 (60.0)	1 (4.6)
Very much	18 (56.3)	10 (47.6)	6 (42.9)	4 (40.0)	19 (86.4)
Days for symptoms to start to improve after phototoxic reaction	*n =* 31	*n =* 21	*n =* 14	*n =* 9	*n =* 18
Mean±SD	2.5 ± 1.6	2.1 ± 1.0	2.6 ± 2.1	2.2 ± 0.8	2.1 ± 1.3
Median (25–75 percentiles)	2 (2–3)	2 (1–3)	2 (1–3)	2 (2–3)	2 (1–2)
Days for all symptoms to go away after phototoxic reaction	*n =* 29	*n =* 21	*n =* 14	*n =* 9	*n =* 19
Mean±SD	5.0 ± 2.9	4.6 ± 1.8	5.1 ± 2.7	5.8 ± 3.4	4.9 ± 2.0
Median (25–75 percentiles)	4 (3–6)	5 (4–5)	4.5 (3–7)	5 (5–6)	5 (4–6)

When asked about their last phototoxic reaction’s effect on their ability to do daily activities, 58% indicated that it affected their ability “very much” (range = 40% Spain to 86% UK). After a phototoxic reaction, it took, on average, 2.1 days for symptoms to improve in Germany and the UK, 2.2 days in Spain, 2.5 days in France, and 2.6 days in Italy. The average time it took for all symptoms to go away after a phototoxic reaction ranged from 4.6 days (Germany) to 5.8 days (Spain) (UK = 4.9 days, France = 5.0 days, Italy = 5.1 days) (Table 3).

Sixty-one percent of the full sample reported at least “some difficulty” in doing chores such as yardwork, gardening, or washing a car for at least 15 minutes when it is sunny outside (range: 52% for Germany − 77% for the UK). In addition, 61% of the full sample reported at least “some difficulty” in going for a walk, run, or bike ride for at least 15 minutes when it is sunny outside (range: 48% for Germany − 77% for the UK). Further, 67% of the full sample reported at least “a little difficulty” being able to run errands and go to stores for at least 15 minutes when it is sunny outside (range: 57% for Germany − 91% for the UK).

### HRQoL, physical, emotional, and psychological well-being

**Fig. 1:**
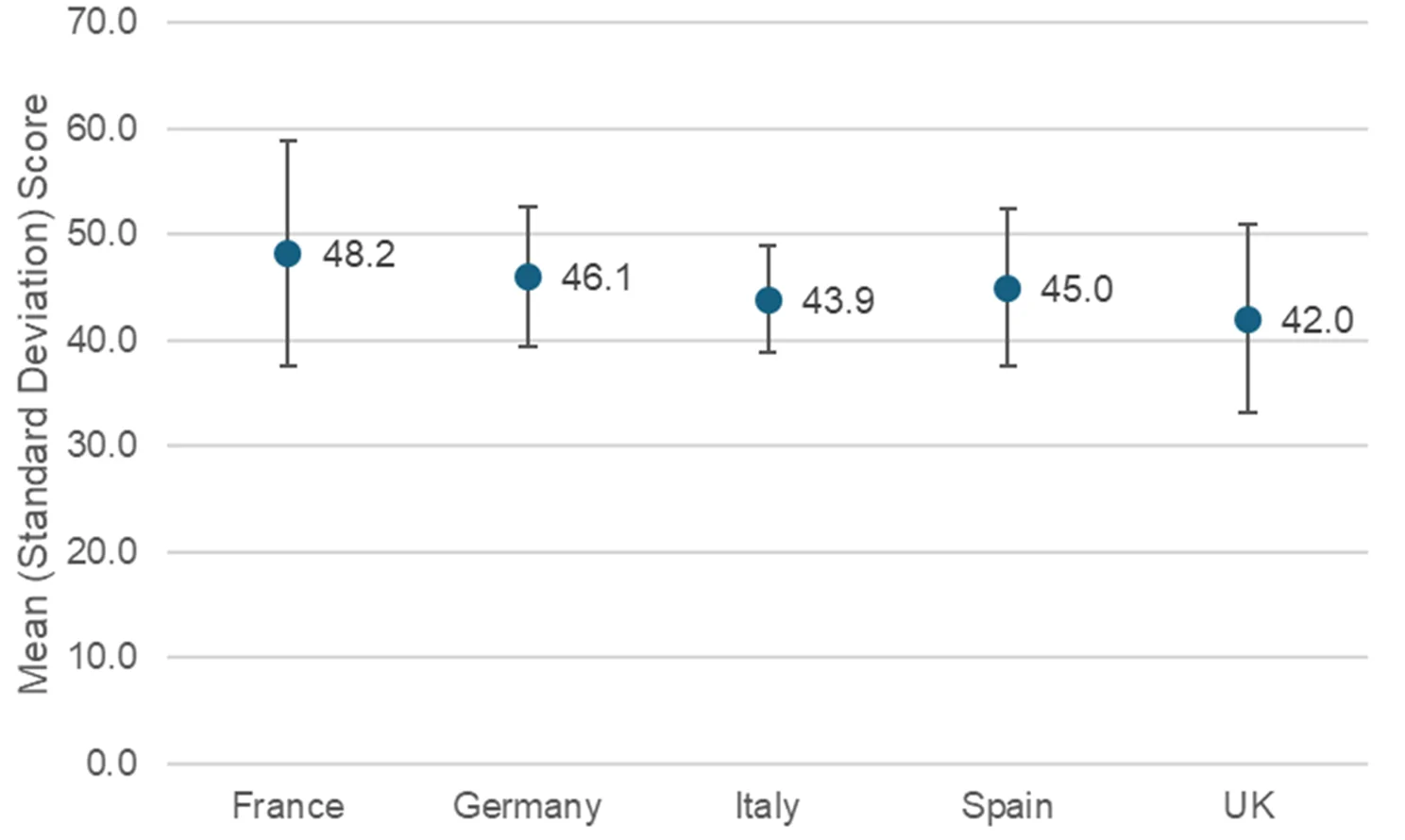
Mean and standard deviation of PROMIS – satisfaction with participation in social roles and activities, by country

The mean T-score on the PROMIS Satisfaction with Social Roles and Activities Short Form − 8a for adults was 45.4 ± 8.8 and 46.7 ± 8.1 for adolescents. More than half (58% of adults and 60% of adolescents) scored more than three points worse than the general population. Within each country, the mean T-score was lowest (worst) in the UK (42.0 ± 8.9) and highest (best) in France (48.2 ± 10.7); mean T-scores in other countries were: 46.1 ± 6.6 (Germany), 43.9 ± 5.0 (Italy), and 45.0 ± 7.4 (Spain) (Fig. 1). In addition, 52, 86, 93, 100, and 86% in France, Germany, Italy, Spain, and the UK, respectively, scored lower (i.e., worse) than the score for the general population, a 50, indicating that most participants had lower satisfaction with social roles and activities than the general population.

The mean T-score on the PROMIS Social Isolation − 4a for adults was 55.3 ± 8.0, while for adolescents it was 47.4 ± 7.5; 68 and 40% of adults and adolescents, respectively, scored higher than 50 (i.e., worse than the general population). The mean T‑score was highest (indicating more isolation) in the UK (56.1 ± 8.8) and lowest (indicating less isolation) in Spain (53.3 ± 5.4). The proportion of respondents scoring above 50 was 64% in France, 67% in Germany and Italy, 70% in Spain, and 82% in the UK. In all countries except Spain (40%), the majority reported a T‑score of 53 or higher, ranging from 61% in France to 68% in the UK.

**Fig. 2:**
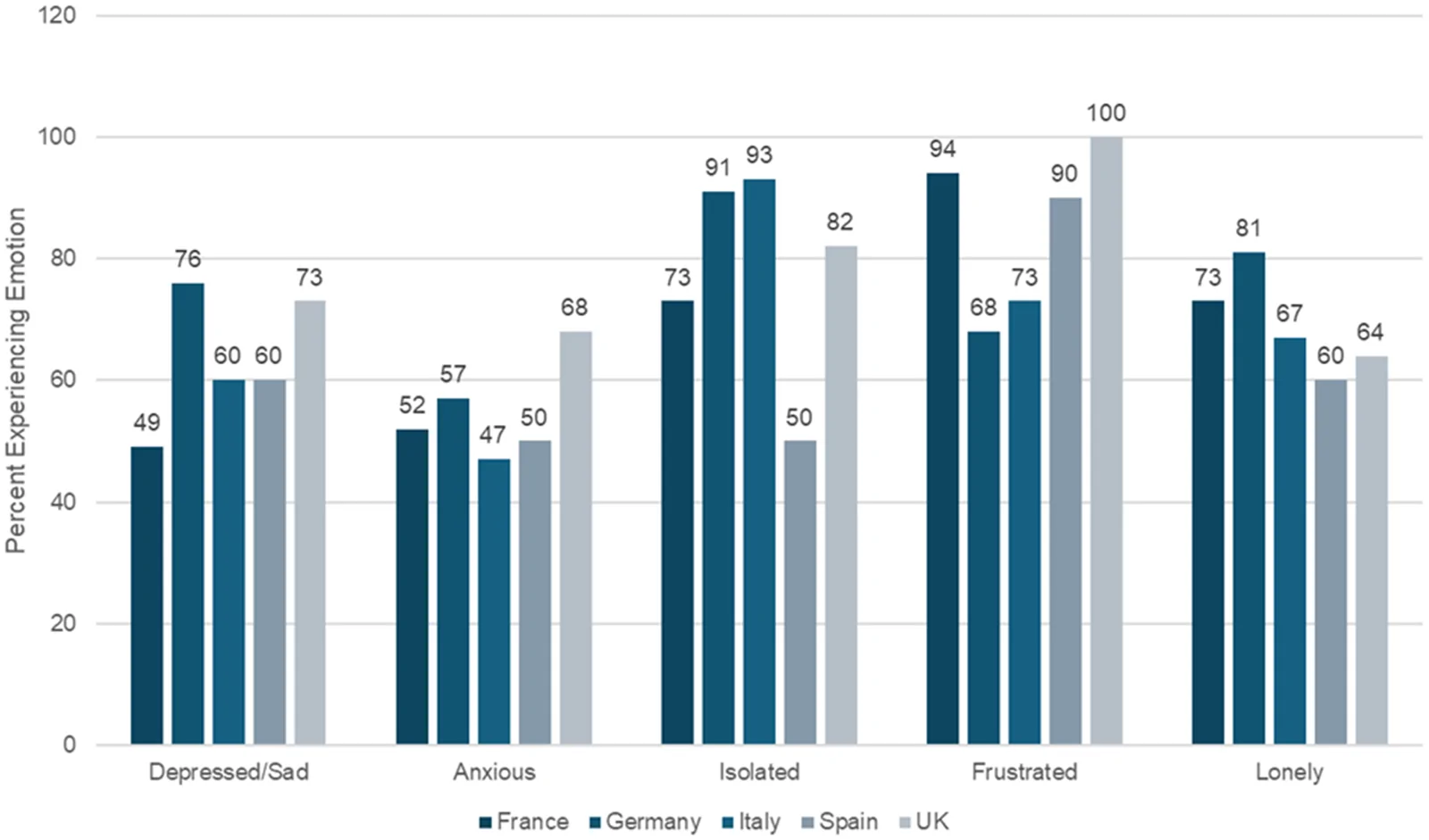
Percent of participants experiencing negative emotions due to EPP, by country

Many adult participants reported experiencing negative emotions, including sadness/depression (66%), anxiety (60%), frustration (86%), loneliness (74%), and isolation (84%). The prevalence of negative emotions in individual countries varied. The percent reporting feeling depressed or sad ranged from 49% (France) to 76% (Germany), anxiety ranged from 47% (Italy) to 68% (UK), isolation from 50% (Spain) to 93% (Italy), frustration from 68% (Germany) to 100% (UK), and loneliness from 60% (Spain) to 81% (Germany) (Fig. 2).

For the KIDSCREEN-27, the mean T-score for the Psychological Well-Being scale was 50 ± 9.0 for adolescents, while for the Peers and Social Support scale the mean T-score was 48.2 ± 14.4, meaning that, on average, their social support was somewhat lower than the general population.

### EPP impact on daily activities and work/school productivity

Forty-one percent of participants said having EPP impacted their ability to do the things they wanted to do in the past 7 days at least “somewhat” (range: 20% for Italy to 58% for France).

At least 60% of participants were currently working for pay (range: 60% Spain to 85% Germany). On average, participants missed less than 4 hours of work per month due to EPP (range: 0 for Spain to 4 hours for France). The impact of EPP on work productivity varied by country: 9% (the lowest) of those in Italy said EPP impacted their work productivity at least “sometimes” compared to 42% in France (the highest).

All 10 adolescents in the study were currently attending school. They missed an average of 3.9 ± 7.2 hours of school due to EPP in the past month (range = 0 to 21 hours). Two of 10 (20%) said that EPP affected their productivity at school at least “somewhat,” while 5 of 10 (50%) said EPP affected productivity at least “a little bit.”

### Healthcare resource use

The total mean number of physician visits in the past 12 months was relatively similar for the UK (5.3 ± 6.0), Germany (5.8 ± 3.9), and France (6.5 ± 7.1), but Italy reported a total of 12.1 ± 11.1 (range: 1 to 40) visits and Spain reported 3.2 ± 1.3 visits (range 1 to 5). Very few ER visits or hospitalizations over the previous 12 months were reported by European participants. One ER visit was reported in France, Spain, Italy, and the UK, in three cases it was for pain from a full reaction (one of those also noted problems with their gallbladder). A single hospitalization was reported in France (for liver problems), Spain (reason not given), and the UK (for pain from a full reaction). None of the participants in Germany reported an ER visit or hospitalization.

The most common medications being used currently for treating, preventing, or managing EPP are vitamin D (75%), non-steroidal anti-inflammatory drugs (11%), anti-depressants (adults only; 24%), beta carotene (14%), iron (9% - three of five individuals with XLP and six of 60 individuals with EPP), and vitamin C (7%). Twenty-four percent are currently receiving afamelanotide (all adults), and 13% (11% of adults and 30% of adolescents) are receiving an investigational drug (of these, 77% [70% of adults and 100% of adolescents] are receiving dersimelagon). Use of afamelanotide differs by country: 0% of participants in either France or Spain are receiving it, while 14% of UK participants, 50% of those in Italy, and 65% of those in Germany are receiving it (Supplemental Table S2). Four percent were also using an opioid for pain relief (such as oxycodone, fentanyl, morphine, hydrocodone) associated with EPP; all those using opioids are from either France or Germany.

Participants were asked based on their experience with all various treatments for EPP or other conditions, how would they prefer to receive their EPP treatment in the future. The majority indicated that they would want to receive a pill (68%; range = 50% in Spain to 87% in Italy). When asked why they prefer their selected mode of administration, the most common reason given was ease of administration (48%) followed by convenience (33%).

## Discussion

In this study of European individuals with EPP, prodromal symptoms were common after ≤ 30 minutes in the sun, often resulting in skin sensitivity or pain and impacting the ability to perform daily activities. When compared with a sample of 164 adults and 33 adolescents from North America (NA) who completed the same questionnaire [23], several findings are notable. First, while European adult participants reported prodromal symptom occurrence slightly less often than NA participants (76% vs 83%), European participants more often reported pain from phototoxic reactions after either indirect (53% vs. 39%) or direct sunlight (69% vs. 64%). Additionally, a slightly higher percentage of European participants reported that phototoxic reactions affect their ability to do daily activities compared with NA participants (58% vs 53%). Fewer European individuals (8.9%) reported liver disease than their NA counterparts (13.2%). However, among those with liver disease, the mean time since their liver disease diagnosis was longer for Europeans (18.2 ± 17.3 years) than for those from NA (8.0 ± 8.3 years).

Results of the current study also demonstrate that EPP imposes a significantly negative burden on the emotional and mental well-being of individuals: 58% of adult participants and 60% of adolescents scored more than three points worse on the PROMIS Satisfaction with Social Roles and Activities than the general population (i.e., <47 points), indicating lower satisfaction with social roles and activities. A large proportion of participants reported that EPP at least “somewhat” impacts their ability to do things they want to do. This is likely due to experiencing symptoms of EPP as well as needing to avoid light exposure to prevent symptoms. Additionally, feelings of sadness, anxiety, frustration, isolation, and loneliness were extremely common. Although prior studies in EPP have noted impairments in HRQoL, they used generic dermatology-specific measures such as the Dermatology Life Quality Index [14]. The current study used EPP-specific measures and was able to highlight more specific aspects that are impacted. However, even considering the potential for more accurate reporting from a disease-specific tool, it is possible there is some underreporting of impacts due to long-term adaptive behaviors and that some participants completed the questionnaire during autumn months.

The impact of EPP on work varied by country. In Italy, 9% said EPP impacted their work productivity at least “sometimes” compared to 42% in France. This is in line with European studies of the impact of other dermatologic conditions, including atopic dermatitis [24], hidradenitis suppurativa [25], chronic urticaria [26], and severe psoriasis [27].

Among the 10 adolescents in the study, 50% indicated at least a little impact on school productivity. In a study of youth (aged 8–18) in the Netherlands and Belgium diagnosed with EPP, 47% reported that they experienced some restrictions in school participation and scored worse on QoL measures of physical and social functioning than matched controls (family, friends, or acquaintances of participant with EPP) [12], reflecting similar impacts on those in school to the current study.

Strengths of this study include the use of a comprehensive questionnaire that included several existing validated measures. Additionally, the study utilized a large sample size from five key European countries. It should be noted that participants’ EPP diagnosis (and other clinical conditions) was via self-report; while the individuals were recruited through patient advocacy groups in each country, diagnoses were not independently confirmed by a physician. It is also possible that participants were more highly motivated and engaged in the management and treatment of EPP than other individuals with EPP. Some questions had recall periods of the past 3 months, 1 month, or 7 days which may have resulted in underreporting of symptoms and impacts if responses were completed during autumn months. As well, participants were not asked what symptoms they associated with a full reaction to compare/contrast with prodromal symptoms. Finally, the study population was primarily of self-reported White European background.

## Conclusions

Europeans with EPP frequently experience prodromal and phototoxic reactions that have a negative impact on their well-being and ability to do daily activities. EPP also appears to negatively impact their emotional functioning, and it contributed to work and school absenteeism. This highlights the need for early diagnosis and effective treatments to reduce the significant burden imposed by EPP.

## Electronic supplementary material

Below is the link to the electronic supplementary material.

Supplementary Material 1

## Data Availability

The data that support the findings of this study are available from the corresponding author upon reasonable request.

## Abbreviations

EPIQ: EPP Impact Questionnaire
EPP: Erythropoietic protoporphyria
ER: Emergency room
HRQoL: Health-related quality of life
HRU: Healthcare resource use
NA: North America
PRO: Patient-reported outcome
PROMIS: Patient-Reported Outcomes Measurement System
UK: United Kingdom

## Footnotes

1 Note: For clear distinction EPP (autosomal erythropoietic protoporphyria) falls under the ORPHA code: 79,278 and XLP (X-linked erythropoietic protoporphyria) uses the ORPHA code: 443,197.

## References

[1] Balwani M, Naik H, Anderson KE, Bissell DM, Bloomer J, Bonkovsky HL, et al. Clinical, biochemical, and genetic characterization of North American patients with erythropoietic protoporphyria and X-linked protoporphyria. JAMA Dermatol. 2017;153(8):789–96. 10.1001/jamadermatol.2017.1557. PMC5710403 28614581

[2] Thapar M, Bonkovsky HL. The diagnosis and management of erythropoietic protoporphyria. Gastroenterol Hepatol (N Y). 2008;4(8):561–66. PMC3096108 21960936

[3] Balwani M, Bloomer J, Desnick R. Adam Mp, Feldman J, Mirzaa Gm, et al editors. Network porphyrias consortium of the NIH-Sponsored Rare Diseases clinical Research. Erythropoietic protoporphyria, autosomal recessive. GeneReviews(R). Seattle (WA), Seattle: University of Washington; 2017. 2012 Sep 27.

[4] Heerfordt IM, Philipsen PA, Lerche CM, Wulf HC. Phototesting in erythropoietic protoporphyria trials: a systematic review. Exp Dermatol. 2023;32(7):1042–47. 10.1111/exd.14809. 37052136

[5] Lecha M, Puy H, Deybach JC. Erythropoietic protoporphyria. Orphanet J Rare Dis. 2009;4(1):19. 10.1186/1750-1172-4-19. PMC2747912 19744342

[6] Elder G, Harper P, Badminton M, Sandberg S, Deybach JC. The incidence of inherited porphyrias in Europe. J Inher Metab Disea. 2013;36(5):849–57. 10.1007/s10545-012-9544-4. 23114748

[7] Whatley SD, Mason NG, Holme SA, Anstey AV, Elder GH, Badminton MN. Molecular epidemiology of erythropoietic protoporphyria in the U.K. Br J Dermatol. 2010;162(3):642–46. 10.1111/j.1365-2133.2010.09631.x. 20105171

[8] Marko PB, Miljković J, Gorenjak M, Povalej P, Kansky A. Erythropoietic protoporphyria patients in Slovenia. Acta Dermatovenerol Alp Pannonica Adriat. 2007;16(3):99–102, 104. 17994169

[9] Lala SM, Naik H, Balwani M. Diagnostic delay in erythropoietic protoporphyria. J Pediatrics. 2018;202:320–3.e2. 10.1016/j.jpeds.2018.06.001. PMC6203604 30041937

[10] Naik H, Overbey JR, Desnick RJ, Anderson KE, Bissell DM, Bloomer J, et al. Evaluating quality of life tools in North American patients with erythropoietic protoporphyria and X-linked protoporphyria. JIMD Rep. 2019;50(1):9–19. 10.1002/jmd2.12052. PMC6850979 31741822

[11] Naik H, Shenbagam S, Go AM, Balwani M. Psychosocial issues in erythropoietic protoporphyria - the perspective of parents, children, and young adults: a qualitative study. Mol Genet Metab. 2019;128(3):314–19. 10.1016/j.ymgme.2019.01.023. PMC6660424 30711301

[12] Kluijver LG, Wensink D, Wagenmakers M, Huidekoper HH, Witters P, Rymen D, et al. Quality of life in children with erythropoietic protoporphyria: a case–control study. J Dermatol. 2024;51(8):1068–78. 10.1111/1346-8138.17348. PMC11484138 38923596

[13] Frank J, Poblete-Gutiérrez P. Delayed diagnosis and diminished quality of life in erythropoietic protoporphyria: results of a cross-sectional study in Sweden. J Intern Med. 2011;269(3):270–74. 10.1111/j.1365-2796.2010.02283.x. 21332583

[14] Holme SA, Anstey AV, Finlay AY, Elder GH, Badminton MN. Erythropoietic protoporphyria in the U.K.: clinical features and effect on quality of life. Br J Dermatol. 2006;155(3):574–81. 10.1111/j.1365-2133.2006.07472.x. 16911284

[15] Wensink D, Wagenmakers MAEM, Wilson JHP, Langendonk JG. Erythropoietic protoporphyria in the Netherlands: clinical features, psychosocial impact and the effect of afamelanotide. J Dermatol. 2023;50(4):445–52. 10.1111/1346-8138.16690. 36579412

[16] Mathias SD, Burke L, Colwell HH, Mensing G, Savage W, Naik H. Development and content validation of novel patient-reported outcome measures to assess disease severity and change in patients with erythropoietic protoporphyria: the EPP impact questionnaire (EPIQ). PROM. 2024;15:17–30. 10.2147/PROM.S438892. PMC10875163 38375415

[17] Mathias SD, Burke L, Colwell HH, Mensing G, Savage W, Naik H. Development and content validation of a sunlight exposure diary in patients with erythropoietic protoporphyria. J Patient Rep Outcomes. 2023;7(1):119. 10.1186/s41687-023-00655-y. PMC10660587 37982964

[18] Ravens-Sieberer U, Auquier P, Erhart M, Gosch A, Rajmil L, Bruil J, et al. The KIDSCREEN-27 quality of life measure for children and adolescents: psychometric results from a cross-cultural survey in 13 European countries. Qual Life Res. 2007;16(8):1347–56. 10.1007/s11136-007-9240-2. 17668292

[19] Robitail S, Ravens-Sieberer U, Simeoni MC, Rajmil L, Bruil J, Power M, et al. Testing the structural and cross-cultural validity of the KIDSCREEN-27 quality of life questionnaire. Qual Life Res. 2007;16(8):1335–45. 10.1007/s11136-007-9241-1. 17668291

[20] HealthMeasures. Intro to PROMIS [Internet]. 2025 Nov 9. https://www.healthmeasures.net/explore-measurement-systems/promis/intro-to-promis.

[21] HealthMeasures. Meaningful change for PROMIS [Internet]. 2025 Nov 9. https://www.healthmeasures.net/score-and-interpret/interpret-scores/promis/meaningful-change.

[22] Terwee CB, Peipert JD, Chapman RS, Lai JS, Terluin B, Cella D, et al. Minimal important change (MIC): a conceptual clarification and systematic review of MIC estimates of PROMIS measures. Qual Life Res. 2021;30(10):2729–54. 10.1007/s11136-021-02925-y. PMC8481206 34247326

[23] Naik H, Balwani M, Colwell HH, Mathias SD, Wheeden K, Norregaard C, et al. The burden of erythropoietic protoporphyria (EPP): results from the EPP life impact and genetic health trajectory (LIGHT) study. J Dermatol. 2025;52(10):1576–83. 10.1111/1346-8138.17897. 40781912

[24] Andersen L, Nyeland ME, Nyberg F. Increasing severity of atopic dermatitis is associated with a negative impact on work productivity among adults with atopic dermatitis in France, Germany, the U.K. and the U.S.A. Br J Dermatol. 2020;182(4):1007–16. 10.1111/bjd.18296. PMC7187138 31260080

[25] Yao Y, Jørgensen A-HR, Thomsen SF. Work productivity and activity impairment in patients with hidradenitis suppurativa: a cross-sectional study. Int J Dermatol. 2020;59(3):333–40. 10.1111/ijd.14706. 31736064

[26] Baudy A, Raison-Peyron N, Serrand C, Crepy MN, Du-Thanh A. Impact of chronic spontaneous or inducible urticaria on occupational activity. Acta Derm Venereol. 2024;104:36122. 10.2340/actadv.v104.36122. PMC11000653 38551378

[27] Meyer N, Paul C, Feneron D, Bardoulat I, Thiriet C, Camara C, et al. Psoriasis: an epidemiological evaluation of disease burden in 590 patients. J Eur Acad Dermatol Venereol. 2010;24(9):1075–82. 10.1111/j.1468-3083.2010.03600.x. 20236205

