# Supplementary material for: Erythropoietic protoporphyria life impact and genetic health trajectory (LIGHT) study in Europe: a cross-sectional online questionnaire
Source: Orphanet J Rare Dis. 2026 Aug 27;21:398. doi: 10.1186/s13023-026-04572-3 (PMC13741743; doi:10.1186/s13023-026-04572-3)
Supplement: Supplementary file 1 [file 13023_2026_4572_MOESM1_ESM.docx]

Supplemental Table 1	Frequency and impact of prodromal symptoms for adult and adolescent participants

| **Characteristic** | **Adolescents**  **(*n* = 10)** | **Adults**  **(*n* = 91)** |
| --- | --- | --- |
| Time in sunlight before having prodromal symptoms, n (%) | *n* = 8 | *n =* 87 |
| ≤ 10 minutes | 2 (25.0) | 24 (27.6) |
| >10 to ≤ 30 minutes | 1 (12.5) | 42 (48.3) |
| > 30 minutes | 5 (62.5) | 21 (24.1) |
| Times in past 3 months experienced prodromal symptoms, n (%) | *n =* 10 | *n* = 86 |
| 0 times | 3 (30.0) | 10 (11.6) |
| 1 to 4 times | 2 (20.0) | 33 (38.4) |
| 5 to 9 times | 2 (20.0) | 9 (10.5) |
| 10+ times | 3 (30.0) | 34 (39.5) |
| Did prodromal symptoms affect ability to do daily activities, n (%) | *n =* 10 | *n =* 88 |
| Not at all | 0 (0.0) | 5 (5.7) |
| A little bit | 0 (0.0) | 20 (22.7) |
| Somewhat | 0 (0.0) | 24 (27.3) |
| Quite a bit | 6 (60.0) | 24 (27.3) |
| Very much | 4 (40.0) | 15 (17.1) |
| How often experience prodromal symptoms before a phototoxic reaction, n (%) | *n =* 8 | *n =* 86 |
| Never | 0 (0.0) | 1 (1.1) |
| Rarely | 0 (0.0) | 0 (0.0) |
| Sometimes | 2 (25.0) | 14 (15.9) |
| Usually | 4 (50.0) | 21 (23.9) |
| Always | 2 (25.0) | 50 (56.8) |

Supplemental Table 2	Treatments and procedures by European country

| **Characteristic** | **France (*n =* 31)** | **Germany (*n =* 20)** | **Italy (*n =* 14)** | **Spain (*n =* 10)** | **UK (*n =* 21)** |
| --- | --- | --- | --- | --- | --- |
| Current treatment for EPP, n (%) |  |  |  |  |  |
| Scenesse (afamelanotide) | 0 (0.0) | 13 (65.0) | 7 (50.0) | 0 (0.0) | 3 (14.3) |
| Beta-carotene (solatene) | 7 (22.6) | 0 (0.0) | 2 (15.4) | 3 (30.0) | 1 (4.8) |
| Oral cysteine | 0 (0.0) | 0 (0.0) | 0 (0.0) | 0 (0.0) | 0 (0.0) |
| Cholestyramine | 0 (0.0) | 0 (0.0) | 0 (0.0) | 0 (0.0) | 0 (0.0) |
| Activated charcoal | 0 (0.0) | 0 (0.0) | 0 (0.0) | 0 (0.0) | 0 (0.0) |
| Cimetidine | 0 (0.0) | 0 (0.0) | 0 (0.0) | 0 (0.0) | 0 (0.0) |
| Neurontin | 0 (0.0) | 0 (0.0) | 0 (0.0) | 1 (10.0) | 0 (0.0) |
| Hematin | 0 (0.0) | 0 (0.0) | 0 (0.0) | 0 (0.0) | 0 (0.0) |
| Plasmapheresis | 0 (0.0) | 1 (5.0) | 0 (0.0) | 0 (0.0) | 0 (0.0) |
| Ursodeoxycholic acid | 0 (0.0) | 0 (0.0) | 4 (30.8) | 1 (10.0) | 0 (0.0) |
| Blood transfusions | 0 (0.0) | 0 (0.0) | 0 (0.0) | 0 (0.0) | 0 (0.0) |
| Anti-depressant | 0 (0.0) | 1 (5.0) | 2 (15.4) | 2 (20.0) | 3 (13.6) |
| NSAID | 2 (6.5) | 0 (0.0) | 2 (15.4) | 1 (10.0) | 5 (23.8) |
| Phototherapy | 1 (3.1) | 0 (0.0) | 0 (0.0) | 0 (0.0) | 1 (4.8) |
| Opioid pain reliever | 1 (3.1) | 3 (15.0) | 0 (0.0) | 0 (0.0) | 0 (0.0) |
| Iron | 5 (15.6) | 0 (0.0) | 0 (0.0) | 3 (30.0) | 1 (4.8) |
| Vitamin E | 0 (0.0) | 0 (0.0) | 0 (0.0) | 0 (0.0) | 1 (5.0) |
| Zinc | 0 (0.0) | 2 (10.0) | 0 (0.0) | 1 (10.0) | 0 (0.0) |
| Vitamin D | 23 (74.2) | 14 (66.7) | 13 (92.9) | 8 (80.0) | 15 (68.2) |
| Vitamin C | 3 (10.3) | 1 (5.6) | 0 (0.0) | 0 (0.0) | 2 (9.1) |
| Investigational drug | 6 (18.2) | 0 (0.0) | 6 (40.0) | 1 (10.0) | 0 (0.0) |
| Bitopertin | 0 (0.0) | 0 (0.0) | 0 (0.0) | 0 (0.0) | 0 (0.0) |
| Dersimelagon | 5 (83.3) | 0 (0.0) | 5 (83.3) | 0 (0.0) | 0 (0.0) |
| None provided/other | 1 (16.7) | 0 (0.0) | 1 (16.7) | 1 (100) | 0 (0.0) |
| Other | 2 (6.1) | 0 (0.0) | 2 (13.3) | 0 (0.0) | 1 (4.6) |
| Procedures for EPP, n (%) |  |  |  |  |  |
| Gallbladder removed | 4 (12.1) | 2 (9.5) | 4 (30.8) | 1 (10.0) | 6 (27.3) |
| Liver ultrasound | 28 (84.5) | 20 (95.2) | 13 (86.7) | 9 (90.0) | 10 (45.5) |
| Liver biopsy | 1 (3.1) | 1 (4.8) | 3 (21.4) | 0 (0.0) | 2 (9.5) |
| Liver transplant | 0 (0.0) | 0 (0.0) | 0 (0.0) | 0 (0.0) | 0 (0.0) |
| Bone marrow transplant | 0 (0.0) | 0 (0.0) | 0 (0.0) | 0 (0.0) | 0 (0.0) |
| Other | 50 (15.2) | 40 (20.0) | 10 (6.7) | 4 (40.0) | 5 (23.8) |

EPP: erythropoietic protoporphyria; NSAID: non-steroidal anti-inflammatory drug; UK: United Kingdom
